# Remote Sensing Image Scene Classification in Hybrid Classical–Quantum Transferring CNN with Small Samples

**DOI:** 10.3390/s23188010

**Published:** 2023-09-21

**Authors:** Zhouwei Zhang, Xiaofei Mi, Jian Yang, Xiangqin Wei, Yan Liu, Jian Yan, Peizhuo Liu, Xingfa Gu, Tao Yu

**Affiliations:** 1 Aerospace Information Research Institute, Chinese Academy of Sciences, Beijing 100094, China; zhangzw@aircas.ac.cn (Z.Z.); mixf@aircas.ac.cn (X.M.); weixq@aircas.ac.cn (X.W.); liuyan@aircas.ac.cn (Y.L.); yanjian19@mails.ucas.ac.cn (J.Y.); yutao@aircas.ac.cn (T.Y.); 2 National Engineering Laboratory for Satellite Remote Sensing Applications, Beijing 100094, China; 3 School of Instrumentation and Optoelectronic Engineering, Beihang University, Beijing 100191, China; liupeizhuo@buaa.edu.cn

**Keywords:** CNN, hybrid classical–quantum neural networks, transfer learning, variational quantum circuit

## Abstract

The scope of this research lies in the combination of pre-trained Convolutional Neural Networks (CNNs) and Quantum Convolutional Neural Networks (QCNN) in application to Remote Sensing Image Scene Classification(RSISC). Deep learning (RL) is improving by leaps and bounds pretrained CNNs in Remote Sensing Image (RSI) analysis, and pre-trained CNNs have shown remarkable performance in remote sensing image scene classification (RSISC). Nonetheless, CNNs training require massive, annotated data as samples. When labeled samples are not sufficient, the most common solution is using pre-trained CNNs with a great deal of natural image datasets (e.g., ImageNet). However, these pre-trained CNNs require a large quantity of labelled data for training, which is often not feasible in RSISC, especially when the target RSIs have different imaging mechanisms from RGB natural images. In this paper, we proposed an improved hybrid classical–quantum transfer learning CNNs composed of classical and quantum elements to classify open-source RSI dataset. The classical part of the model is made up of a ResNet network which extracts useful features from RSI datasets. To further refine the network performance, a tensor quantum circuit is subsequently employed by tuning parameters on near-term quantum processors. We tested our models on the open-source RSI dataset. In our comparative study, we have concluded that the hybrid classical–quantum transferring CNN has achieved better performance than other pre-trained CNNs based RSISC methods with small training samples. Moreover, it has been proven that the proposed algorithm improves the classification accuracy while greatly decreasing the amount of model parameters and the sum of training data.

## 1. Introduction

In recent times with the rocketing progress of the earth observation capability and the artificial intelligent technology, the progression and innovation of remote sensing (RS) are more important than ever. RSI data processing is now developing towards RSI the age of big data, which relies on data model by data-driven intelligent analysis [1] (refer to Table 1 for all acronyms that are used throughout the paper). Machine learning (ML) plays an essential role in extracting underlying features or a probability distribution of patterns in RSI datasets, such as damage prediction, target recognition or image classification in hitherto unknown domains [2]. In particular, deep learning (DL) has proven to be a technological innovation and a historic milestone in many fields [3]. It emphasizes neural networks (NNs) involving multiple hidden layers, which use feature representations learned exclusively from the data, instead of handcrafted features that are designed based mainly on domain-specific knowledge. DL has widely used in various research topics, for example, data analysis tasks, including image segmentation, image target detection, and image classification.

Various deep learning models have been developed with outstanding performance for image classification on RSI datasets in multiple applications. Recently, significant progress has been made in the methods of RSISC. Early studies involving RSISC in CNN were transplanted from natural image data, which led to the increasing of the difficulty of the RSISC task in complex RSIs [4]. Since then, researchers havededicated to replace hand-engineered features with trainable multilayer networks, and several deep learning models have demonstrated impressive feature representation capability, applicable across a wide range of domains, including RSISC [5]. Deep learning algorithms have opened up an entirely novel frontier of learning algorithms including CNN techniques that have been adopted in the RSISC [6]. CNNs can extract hierarchical and insightful features automatically from the massive size of image data [7].

In recent years, there has been significant progress in RSISC methods using CNNs [2,3,8]. Despite the extraordinary achievement that CNN-based methods have enabled in RSISC, the models of the network construction remain a huge challenge, which is significant for the efficiency of the CNN models [7]. CNN architecture construction requires skill in both DL methodology and professional expertise [9]. In practice, handmade architecture design is complicated and fallible; moreover, the requirement of number of expertise and professionals in both DL and the investigated domain is often unrealistic in practical applications.

In this context, most studies on CNN-based RSISC have utilized networks that are pre-trained on different networks, for instance AlexNet [10], VGGNet [11], GoogleNet [12], and ResNet [13], standing out among numerous DL-based methods due to closely related powerful features extraction performance. Since both assignments involving RSIs data and the pre-trained networks can easily be operated for transferring in RSISC. Although, RSIs differ significantly in terms of spectral resolution, spatial resolution, and radiometric resolution, etc. [14]. Researchers have used pre-trained CNN models as features extractors to extract high semantic features. These methods have limitation: It has no advantage if significant domain differences exist between source and target datasets. 

Fine-tuning CNN-based models is an effective tool for RSISC. However, such approaches still have limitations in three perspectives related to datasets, models, and labels. These limitations are discussed below.

Firstly, the dataset plays an important role in advancing RSISC. The Size-Similarity Matrix of dataset determines the choices of the pre-trained model. This matrix classifies the strategies based on the size of the dataset and its similarity to the dataset in which the pre-trained model was trained. Due to the matrix transformation conducted in the fully connected layers, the dataset must be transformed into a certain fixed size. The most common approach is to crop and interpolate the original image, which inevitably results in a loss of fine information from the original RSIs. Unfortunately, either of these approaches can be detrimental to the performance of RSISI. Therefore, the selection of the pre-trained models depends on the dataset’s size and size-similarity. The RSIs dataset must be transformed into a special fixed size [15]. 

Secondly, from a modeling perspective, most pre-trained models can achieve excellent classification performance. However, these models also have some limitations. The learned feature may be not entirely satisfactory for the properties of target datasets, and pre-trained treatments applied between heterogeneous networks require manual manipulations of layer combinations, depending on type of task. The RSISC task, in particular, requires a massive tuning procedure to achieve optimal classification performance. 

**Figure 1:**
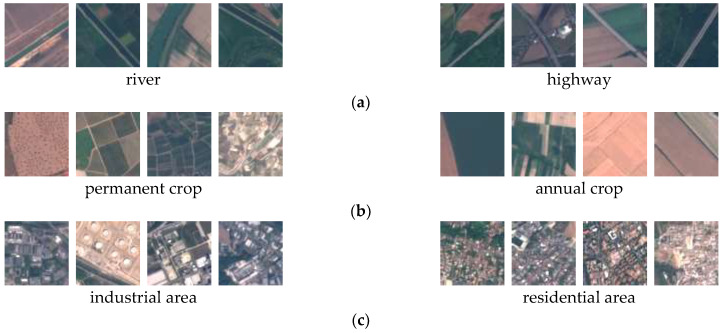
Limitations of RSISC, which include (**a**) low type separability, (**b**) complex variance of scene scales, (**c**) coexistence of multiple objects. The sources are from Eurosat dataset [17].

Thirdly, many scene images with identical exteriors have different but correlated scene contents, which can lead to the label paradox. Because an RSI contains various objects, such as water, mountains, bare land, buildings, etc., which may be covered in a residential scene as shown in Figure 1, the variance and complex spatial distributions of the scene contents cause the diversity of RSISC. Because of the different varieties in the exteriors of ground objects within the similar semantic information, traditional methods of RSISC often fail to achieve satisfactory classification accuracy. In addition, low scene category separability has been caused by the presence of the same scene contents within different scene categories or high semantic superimposition between different scene types. Another important difficulty of RSISC is the large variance of object scales caused by sensor imaging altitude variation. Furthermore, the label distribution of ground objects in datasets is irregular and often unavailable in the original training dataset, which is typically caused by resembling the sample dataset that has different but correlated labels, as can be seen in Figure 1 [16].

Both RSISC and quantum computing are emergent techniques that have the potential to transform the research and application of RS. Quantum computing can provide significant advantages in terms of improving the performance of classification techniques. The great potential of quantum computing in ML has been actively investigated. In specific cases, traditional machine learning tasks can be improved with exponential acceleration when it operates on a quantum computer [18,19]. The Harrow–Hassidim–Lloyd (HHL) algorithm is a quantum computing that has been successfully implanted in conventional ML theories and topics, such as data mining, artificial neural networks, computational learning theory, etc., as shown in [20,21,22,23,24]. HHL-based algorithms are based on the quantum phase estimation algorithm, which operates in a high-depth quantum circuit [25]. To bypass this strict requirement of hardware, classical–quantum hybrid algorithms containing a low–depth quantum circuit, for instance, the variational quantum eigensolver (VQE) and the quantum approximate optimization algorithm (QAOA) have been suggested [26,27]. The idea of a hybrid algorithm is to divide the problem into two components, either of which can be operated on a quantum and a classical computer separately. Cai verified the feasibility of a K-means algorithm on a quantum computer [28]. Otgonbaatar proposed a parameterized quantum circuit (PQC) with only 17 quantum bits for classifying a two-label Sentinel RSI dataset. Quantum-based pseudo-labelling for hyperspectral imagery classification is demonstrated by Shaik [22]. Noisy intermediate-scale quantum (NISQ) devices are an advanced quantum computing technology providing solutions for large-scale and complex practical quantum computation, such as solving high-complexity problems or supporting ML algorithms. An NISQ device is termed a variational quantum circuit (VQC) when it has parameterized quantum gates and parameterized quantum circuit. Sometimes, the VQC is a supervised learning algorithm in which quantum neural networks (QNNs) are trained to perform a classification mission. 

A transformer-based model is one of the successful approaches of DL and more efficient than other traditional CNN models in a variety of downstream applications. Transformer-based concepts in in the context of quantum machine learning consist mainly of three variants: classical–quantum (CQ), quantum–classical (QC), and quantum–quantum (QQ) [23]. The CQ concept can achieve better result in comparison with other transformer-based concept tests and classical–classical concept tests. Therefore, the CQ concept has potential with currently available quantum computers. 

The purpose of this paper is to investigate the feasibility and the efficiency of the hybrid classical–quantum transferring CNN model for RSISC. Our study aims to demonstrate that hybrid classical–quantum transferring CNN models can significantly enhance the efficiency and performance of RSISC with small training samples. We focus on the hybrid models scenario, where tensor quantum circuits and CNNs can be jointly trained to achieve RSISC. With the advent of NISQ devices, CQ transfer learning has become particularly attractive, since, with the application of a classical–quantum neural network with large input images, it is possible to efficiently extract highly informative features with a VQC. It is advantageous, since it makes the application of the potential of quantum physics theory–superposition and entanglement, coordinated with the mature methods of classical ML—possible. Accordingly, tensor quantum circuits can be regarded as common quantum feature extractors, which can mimic famous classical networks that are often used as pre-trained networks.

The transfer learning approach in the quantum domain has been largely unexplored, with only very few applications, such as modeling quantum Many-Body Systems (MBSs), associated with classical autoencoders to a quantum Boltzmann machine, and initializing variational quantum networks. In this paper, an innovative, self-designed, and systematic theory, through a concept of tensor quantum circuits and pre-trained networks, is proposed.

We present a model-theoretic approach and provide proof-of-principle examples for practical implementation through numerical simulations. Additionally, we test our model experimentally on physical quantum processors, successfully conducting RSISC with a hybrid CQ system.

The remaining sections of this work are introduced as follows: Section 2 introduces some notations about hybrid classical–quantum networks. Section 3 outlines the architecture of the hybrid classical–quantum transferring CNN. Section 4 reports the evaluation and results of our model. Finally, this paper is concluded in Section 5. 

## 2. Hybrid Classical–Quantum Networks

### 2.1. Quantum Encoding

The first step is the transformation of the classical image dataset into quantum state; the process denotes the quantum encoding (QE). Most QE methods can be seen as parameterized circuits acting on initial states; the parameters in the parameterized circuits are determined via classical image data. Generally, QE methods can be divided into basis encoding, amplitude encoding, angle encoding, instantaneous quantum polynomial(IQP) encoding, and Hamiltonian evolution ansatz encoding [29,30,31,32]. IQP encoding can be achieved more easily than the other QE methods, so it is applied to our proposed algorithm. Then, IQP quantum encoding describes the following quantum state as Equation (1). *R_Y_*(•) denotes Pauli rotation *Y* gate.

(1) $$\left({⊗}_{i=1}^{4}R_{Y}\left(\pi x_{i}\right)\right){|\left.0\right\rangle }^{⊗4}=[\begin{array}{c} \cos\left(\pi x_{1}\right)\\ \sin\left(\pi x_{1}\right) \end{array}]⊗[\begin{array}{c} \cos\left(\pi x_{2}\right)\\ \sin\left(\pi x_{2}\right) \end{array}]⊗[\begin{array}{c} \cos\left(\pi x_{3}\right)\\ \sin\left(\pi x_{3}\right) \end{array}]⊗[\begin{array}{c} \cos\left(\pi x_{4}\right)\\ \sin\left(\pi x_{4}\right) \end{array}]$$

### 2.2. Variational Quantum Circuits

VQCs usually can define a subcircuit, which is a basic circuit architecture where complex VQCs can be constructed through repeating layers. Circuits consists of multiple rotating gates as well as CNOT gates that entangle each qubit with its neighboring qubit. We also need a circuit to encode the classical data onto the quantum state, so that the output of the measurement is related to the input. In this case, we encode the binary input onto the qubits of the corresponding order. 

VQCs can define a quantum layer like the classical neural network. Furthermore, arbitrary VQCs can be demonstrated as below:

(2) $$L=U\left(w\right)|\left.x\right\rangle$$

where w denotes variational parameters, a unitary operation is achieved, acting on the input state |x> of n_q_ quantum subsystems. 

The depth d of a VQC is a superposition of different quantum circuits and matches the product of various parameterized unitary operations with different weights:

(3) $$L=L_{d}\circ \ldots L_{2}\circ L_{1}$$

On other hand, we can measure the expectation values of n_q_ local observables ẑ = [ẑ_1_, ẑ_2_, … ẑ_n_] for the extraction of a classical output y. The process is known as a measurement layer, which maps a quantum state to a classical dataset:

(4) $$Μ∶=\left\langle x|\hat{z}|x\right\rangle$$

The initial quantum layers and the final measurement state in the quantum circuits can be globally written as follows:

Q = M × L × ε (5)

### 2.3. Tensor Quantum Circuits

The VQC algorithm is limited to parameters adjustment, using the quantum computer as feedback circuits to adjust the parameters in the parameterized circuits to optimal values. It can only optimize the structure of the fixed circuit; it cannot change its own structure, nor increase the number of entanglement operations, nor change the entanglement characteristics expressed, so the algorithm still means the quantum chip entanglement operation. For complex Hamiltonian encoding, such as correlated systems, this common method does not performs well.

**Figure 2:**
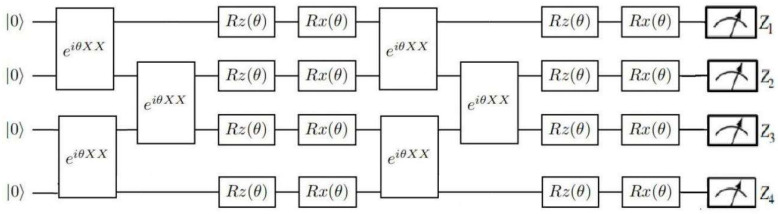
An illustration of tensor quantum circuits.

Tensor quantum circuits adopt the classical quantum hybrid algorithm. The classical part adopts the form of the tensor network to process the higher order tensor into the form of multiple lower order tensor compressors. The quantum part adopts VQC to adjust the parameters. Tensor quantum circuits consist of n qubits and consisting of k layers. Parameterized e^iθX⊗X^ gates are between each neighboring qubit in each layer, followed by a series of single-qubit parameterized Z and X rotations, as can be seen in Figure 2.

## 3. Hybrid Classical–Quantum Transferring CNN

**Figure 3:**
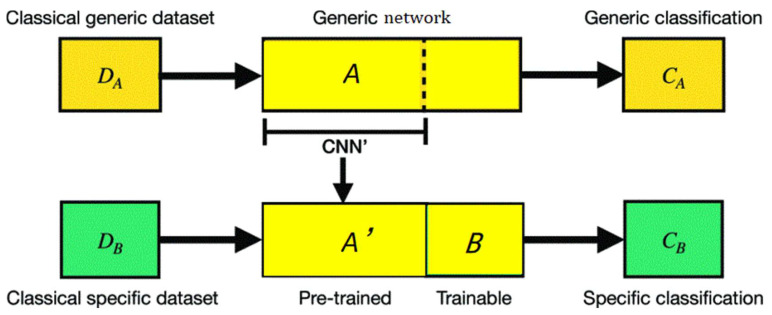
A hybrid classical–quantum transferring CNN for RSISC. ResNet network is utilized as features extraction and tensor quantum circuits are applied as a fully connected layer for RSISC.

In the following section, we introduce the concept of transferring prior “knowledge” from classical to quantum. A hybrid classical–quantum transferring CNN for RSISC proposal on the basis of two networks A and B is defined in Figure 3. 

A hybrid classical–quantum transferring CNN for RSISC proposal (as shown in Figure 3). The pre-trained network A on a dataset D_A_ for a task C_A_ is CNN’. Then, the final layers are removed. The reduced network A’ can be taken as feature extractor. A new trainable network B can integrate closely the pre-trained network A’. The parameters of network B can adjust finely on the specific dataset.

D_A_ = ImageNet: ImageNet Large Sclae Visual Recognition Challenge (ILSRC) with many classes [33].

A = ResNet34: a pre-trained residual neural network [13].

C_A_ = Image classification.

A’ = ResNet34 a residual neural network without the final layer.

D_B_ = RSI datasets 

B = Q = L_4→2_ × Q × L_512→4_: i.e., 4-qubit tensor quantum circuits and outputs.

C_B_ = RSISC.

The strategy of feature extraction is much more general than what we needed in this work. In the context for transfer learning, the reduced pre-trained network A’ is interpreted as a feature extractor, after removing the final layer of A. A’ can produce features that are not problem-specific. In a hybrid structure, network A is classical, and the network B is quantum.

Nowadays, classical hybrid transfer learning is perhaps composed of the efficient and mature tools of DL in the current technological era of ML. It is commonly validated through the successful ML algorithms, especially for RSISC.

The quantum circuit model in Equation (6) demonstrates the concept. We assume quantum circuits of 4 qubits and utilize the following model:

(6) $$\tilde{Q}=L_{4\to 2}\times Q\times L_{2\to 4}$$

where L_2→4_ implies residual networks using the activation function, tanh φ = tanh, Q is VQC, and L_4→2_ denotes residual networks without activation function. The strategy of quantum encoding establishes links between the image dataset input x and its quantum state |x>. The chosen embedding map from the classical image dataset input vector can be written as Equation (8):

(7) $$\varepsilon \left(x\right)={⨂}_{i=1}^{4}\left(R_{y}\left(\pi x_{i}\right)H\right){\left.|0\right\rangle }^{⨂4}=[\begin{array}{c} cos\left(\pi x_{1}\right)\\ sin\left(\pi x_{1}\right) \end{array}]⨂[\begin{array}{c} cos\left(\pi x_{2}\right)\\ sin\left(\pi x_{2}\right) \end{array}]⨂[\begin{array}{c} cos\left(\pi x_{3}\right)\\ sin\left(\pi x_{3}\right) \end{array}]⨂[\begin{array}{c} cos\left(\pi x_{4}\right)\\ sin\left(\pi x_{4}\right) \end{array}]$$

(8) $$L\left(x\right):\left.|x\right\rangle \to \left.|y\right\rangle ={K⨂}_{i=1}^{4}R_{y}\left(\pi x_{i}\right)\left.|x\right\rangle$$

where H denotes a single Hadamard gate. The quantum circuits consist of 4 variational circuits. And K indicates an entangling unitary operation of 3 CNOT gates. The model in the red frame demonstrates CNOT gates and 3 rotation gates R_X_, R_Y_, R_Z_. The CNOT gates force quantum entanglement between 2 quantum circuits, allowing the qubits from the circuits to be entangled. 

Then, the measurement operators should be projected by measuring the expectation values of 4 observables, locally estimated for each qubit:

(9) $$\hat{z}=\left[{\hat{z}}_{1},{\hat{z}}_{2},{\hat{z}}_{3},{\hat{z}}_{n}\right]$$

(10) $$Μ:z=\langle x\left|\hat{z}\right|x\rangle$$

**Figure 4:**
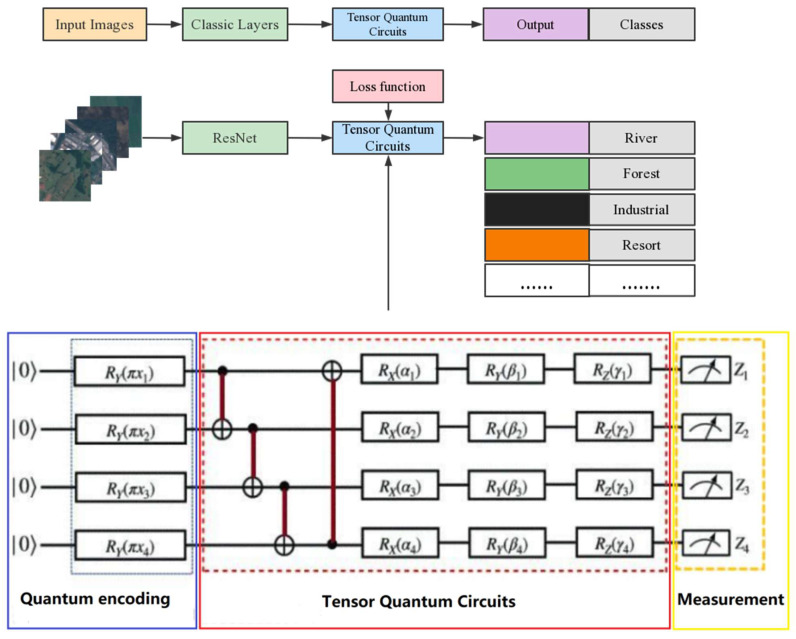
(**Top**): general scheme for the hybrid classical–quantum transferring CNN for RSISC. (**Middle**): detailed scheme for classifying the scene image dataset. (**Bottom**): architecture of the tensor quantum circuit with inputs of 4 qubits. R_x_(•), R_y_(•) and R_z_(•) separately denote Pauli rotation X, Y, Z gates.

In the final classification stage, the cost function was used as cross entropy and via a LogSoftMax layer. The flowchart of our proposed method is shown in Figure 4, and numerical simulations were conducted using the PyTorch interface and PennyLane software.

## 4. Evaluation an Results

In this section, we assess the hybrid classical–quantum transferring CNN in terms of performance gains for RSISC.

### 4.1. Data Profile

**Figure 5:**
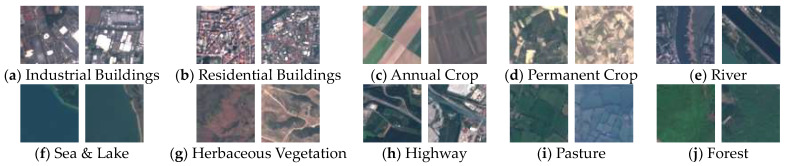
This outline shows all sample images of all 10 categories covered in the EuroSAT dataset. The image size has 64 × 64 pixels. Each category contains 2000 to 3000 images. In sum, the dataset has 27,000 geo-referenced images [17].

**Figure 6:**
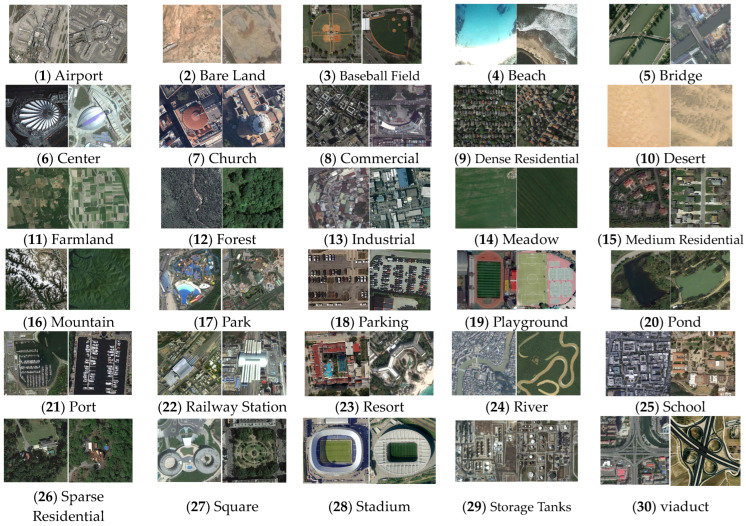
This outline shows all sample images of all 30 categories covered in the AID dataset. The image size has 600 × 600 pixels. Each category contains 220 to 420 images. In all, the dataset has 10,000 georefenced images [34].

Our proposed method was evaluated using two challenging RSI datasets: the EuroSat dataset and the Aerial Image dataset (AID) [17,34]. The EuroSat dataset contains images taken from the Sentinel-2 satellite, categorizing the ground objects into 10 distinct land cover categories. The collection includes approximately 27,000 images divided across 10 classes, with patches measuring 64 × 64 pixels. The data were originally hyperspectral images captured with 13 spectral bands, but we used only RGB channels. The AID dataset includes 10,000 images of size 600 × 600 pixels, classified into 30 scene classes, with varying numbers of images for each label ranging from 220 to 420. The ground resolution also varies from approximately 8 m to 0.5 m per pixel. Figure 5 show examples of the EuroSAT dataset and Figure 6 shows examples of the AID dataset.

### 4.2. Evaluation Criteria

There are two extensively used evaluation criteria in RSISC: overall accuracy (OA) and confusion matrix. OA is an evaluating indicator of the classifier’s performance on the whole test data set and is defined as the sum of accurately classified samples divided by the sum of tested samples. It is a commonly used to evaluate the performance of RSISC. The confusion matrix is an informative table used to analyze all the errors and confusions between different classes, generated by comparing the performance of correct and incorrect classification of each single classifier. 

The definition of the calculation of OA can be expressed as follows:

(11) $$OA=\frac{\sum_{i=1}^{n}\sum_{j=1}^{k}P_{ij}}{T}$$

where P_ij_ is the correct prediction of each single label, and n, k represents the sum of each label and the sum of labels. T is the sum of test dataset.

### 4.3. Experimental Setup

We split two different RSI datasets in different training/test ratios (10/90 ratio and 20/80 ratio) class-wise. The RGB images vast majority of all aerial and RSI datasets. For performance measurement, we compare with the performance of the proposed algorithm, the Bag-of-Visual-Words (BoVW) approach with scale-invariant feature transform (SIFT) features, a trained support vector machine (SVM) and other deep learning algorithms for accuracy evaluation. Furthermore, we trained a shallow CNN, a ResNet50, and a GoogleNet model on the training dataset [33,35,36,37]. In addition to the comparison with our proposed model, it is also compared with existing CQ models available in the literature, namely: CNN-QNN end-to-end model [24] and QCNN [38]. 

In our proposed method, we trained a ResNet34 model and the tensor quantum circuit with the model for 60 epochs over the training dataset, with a quantum depth of 6 and an initial learning rate of η = 0.0006. The model was validated with respect to the test dataset after each epoch, and overall classification on the datasets was calculated. 

We used many commonly used RSISC algorithms for instance, ResNet50 [13], DCNNs [39], AlexNet [40], and VGG-VD16 [10], as criteria to assess the performance improvement of our proposed method. These models were pre-trained on the ImageNet dataset and then fine-tuned to adapt them to the RSIs. For the open-source AID dataset [34], the training:test ratios were set to 20%/80% and 50%/50%.

### 4.4. Performance Comparison

Furthermore, in order to further verify the efficiency and feasibility of the proposed algorithm in this paper, the proposed algorithm was also compared with other RSISC algorithms for experimental analysis and validated on the validation set. The results show that the proposed algorithm achieved the best classification result (%), as shown in following Algorithm 1 procedures.
**Algorithm 1.** Comparation with other RSISC algorithms.**1. INPUT:** RSI dataset as training data, rest RSI dataset as test data. **2. OUTPUT:** Generate the predicted category labels. 3. Prepare a ResNet 34 network with the autoencoder. 4. Encode the RSI dataset. **5. Traning**: a 4 qubit tensor quantum circuit. 6. Feed the RSI dataset to the tensor quantum circuit. **7. Testing:**
8. Feed the rest RSI dataset to the tensor quantum circuit. **9. STOP ALGORITHM.**

As can be seen in Table 2, we can analysis the performance of different approaches on the EuroSAT RGB dataset using various training:test splits. We evaluated four frequently used methods, including BovW, CNN, ResNet50, and GoogleNet, as baselines to assess the performance improvement of the hybrid classical–quantum transferring CNN. We used these pre-trained models on the ImageNet dataset and fine-tuned them on the EuroSAT dataset. For ResNet50 and GoogleNet, we replaced the flattened layer, maintaining the original image sizes. The proposed method exhibited better classification accuracy than other methods and significantly reduced model complexity.

To quantitatively evaluate the performance of our proposed method, we adopted the OA and confusion matrix as the evaluation metrics. OA is recorded as the number of correctly classified samples divided by the total number of samples. In the confusion matrix, each column denotes the predicted results, and each row denotes the actual ground objects of the class data. It can display the distribution of each class and can be recommended for the analysis of misclassification results between different classes.

**Figure 7:**
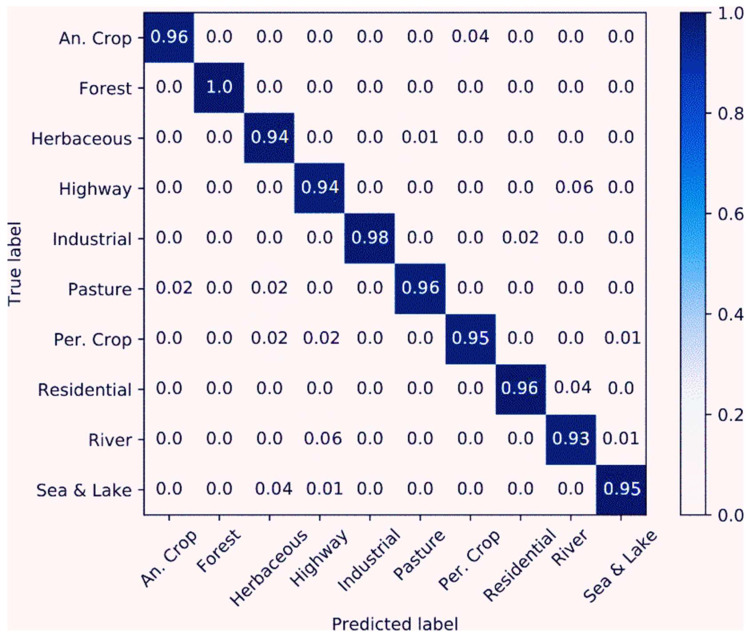
Confusion matrix of the proposed method on the EuroSAT dataset in a training and a test set (10/90 ratio) using RSIs in the RGB color space.

**Figure 8:**
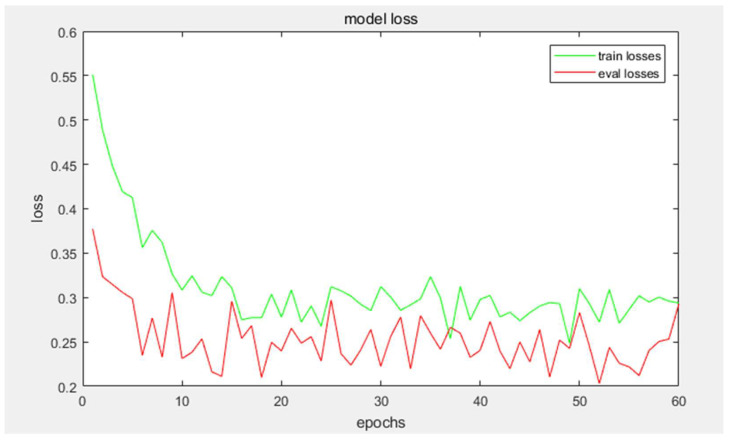
Relation between the loss function and training steps of the proposed method.

**Figure 9:**
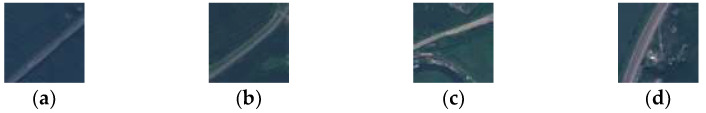
The images misclassified as others. (**a**,**b**) The images of highways misclassified as rivers. (**c**,**d**) The images of rivers misclassified as highways.

Figure 7 present the evaluation of the proposed method on the EuroSAT dataset in a training and a test set (10/90 ratio). The images of river, highway and herbaceous vegetation misclassified easily as others. As can be seen in Table 2, all CNN algorithms surpassed the BoVW method and the classification results of whole deep CNNs performed better than the classificatfigureion results of shallow CNNs. Nevertheless, the proposed method achieved a classification result of up to 95.81% in a training and a test set (10/90 ratio) for the EuroSAT RSISC. The optimization process is demonstrated in Figure 8. As can be seen in Figure 9, the images of two scene labels are alike, resulting in imperfect classification results compared with other labels.

Table 3 shows the resulting classification accuracies for the best performing DL models CNN models, GoogLeNet, ResNet50, and the proposed method. In experiments with the GoogLeNet, ResNet50, CNN models, and the proposed method, all models were pre-trained on the EuroSAT dataset. For a better comparison of performance of all fine-tuning models, we trained the last layer with a learning rate between 0.01 and 0.0001. Using the proposed method, we achieved a classification accuracy about 17% better than the other pre-trained model, GoogleNet, which had been trained on the EuroSAT dataset in the same training:test dataset ratio setting. 

Furthermore, we compared the proposed algorithm with other pre-trained CNN-based classification methods for different training:test ratios on the RSI dataset. As shown in the table below, the most traditional pre-trained CNN models can achieve under 95% for the training ratios of 20%. The hybrid classical–quantum transferring CNN achieved higher accuracy than other pre-trained CNN-based classification methods. In Table 3, the traditional pre-trained models which achieved high classification accuracy use million RSIs from satellite and aerial platforms and cover a huge amount of scenes and objects around the world, for instance, RingMo [41]. The proposed algorithm can also enhance the classification result for the large scale of input images and performs better than all the other algorithms under the training ratio of 20% and 50%, and “*” indicates the best result among all methods. The best classification results can be obtained with the algorithm that achieves 97.33% and 98.82% for the training ratios of 20% and 50%, respectively. Our proposed approach exhibits the performance of the most advanced methods, * indicates the best result among all methods.

**Table 3:** Contrast of the classification accuracies (%) of different training-test ratios on the AID dataset (training ratio = 20% and 50%).

Method	20/80	50/50
VGG-VD16 [34]	86.59	89.64
GoogLeNet [34]	83.44	86.39
AlexNet + MSCP [42]	88.99	92.36
VGG-VD16 + MSCP [42]	91.52	94.42
AlexNet + SPP [43]	87.44	91.45
RADCNet [44]	88.12	92.53
AlexNet + SAFF [45]	87.51	91.83
VGG-VD16 + SAFF [45]	90.25	93.83
AlexNet + RIR [46]	91.95	94.56
VGG-VD16 + RIR [46]	93.34	95.57
ResNet50 + RIR [46]	94.95	96.48
DCNN [40]	90.82	96.89
CBAM [47]	94.66	96.90
Two-Stream Fusion [48]	92.32	94.58
RTN [49]	92.44	_
GCFs + LOFs [50]	92.48	96.85
CapsNet [51]	91.63	94.74
ARCNet [52]	88.75	93.1
SCCov [53]	93.12	96.1
KFBNet [54]	95.50	97.40
GBNet [55]	92.20	95.48
MG-CAP [56]	93.34	96.12
EAM [57]	94.26	97.06
EAM [57]	93.64	96.62
F^2^BRBM [58]	96.05	96.97
MBLANet [59]	95.60	97.14
GRMANet [60]	95.43	97.39
IDCCP [61]	94.80	96.95
MSANet [62]	93.53	96.01
CTNet [63]	96.25	97.70
LSENet [64]	94.41	96.36
DFAGCN [65]	_	94.88
MGML-FENet [66]	96.45	98.60
ESD-MBENet-v1 [67]	96.20	98.85 *
ESD-MBENet-v2 [67]	96.39	98.40
SeCo-ResNet-50 [68]	93.47	95.99
RSP-ViTAEv2-S [69]	96.91	98.22
ISP (ViT) [70]	96.24	97.95
ISSP (ViT) [41]	95.82	97.98
RingMo (ViT-200 W-200 E) [71]	96.54	98.38
ISP (Swin) [70]	96.24	98.03
ISSP (Swin) [72]	96.54	97.95
RingMo (Swin-200 W-200 E) [71]	96.90	98.34
**Ours**	**97.33** *	**98.82**

We have implemented these networks using a neural network library named Pytorch and a cross-platform Python library for quantum computing named PennyLane [39,73]. The experiments have been operated on a computer graphics workstation equipped with a single AMD Ryzen Threadripper PRO 3945WX 12-Cores CPU and a single NVIDIA GeForce NVIDIA RTX A5000 24GB GPU.

In this section, we detail various limitations and challenges of this study and how they could potentially be avoided.

1. Lack of access to high-quality, standardized quantum datasets. More work is needed to develop such datasets to properly benchmark our proposed model.
2. Limited types and amount of remote sensing image (RSI) samples currently used. More RSI data from different satellite sources should be collected.
3. More methods for tensor network-based feature extraction should be explored to improve the interpretability and performance.
4. Further research into CQ transferring CNN architectures with sharp priors is needed, as these can help avoid issues like barren plateaus. Architectures like quantum graph neural networks and quantum recurrent neural networks show promise [74].
5. A theory of “quantum geometric deep learning” could help systematically design architectures suited for different quantum datasets, by encoding appropriate symmetries and physical principles.

In summary, the main limitations involve access to high-quality quantum data, limited diversity of current RSI datasets, and the need for better QCNN architectures. Key future work involves developing standardized quantum datasets, collecting more RSI data, exploring tensor network feature extraction, and establishing a theory of quantum geometric DL.

## 5. Conclusions

Over the past decade, rapid development has provided us with massive remote sensing datasets for intelligent earth observation using RSIs. Nevertheless, the lack of publicly available “big data” of RSIs seriously limits the development of innovative methods, especially traditional DL methods. This work first presents the background of VQC and tensor quantum circuits, and a hybrid classical–quantum transferring CNN applied to RSISC has been proposed. The main advantage of the method in this work is that it allows the input images to be of various shapes and sizes and dispense with the resizing of such images prior to processing. It can hold most key features in RSIs, which is mostly beneficial to conclusively achieving better classification results. In comparison to other deep learning methods with the same number of training samples, the proposed method has achieved amazing scene classification results. Data annotation must be done manually by skilled professionals in the area of RSISC. When a RSI dataset is massive, data annotation can become more complicated because of the huge diversities and variations in RSIs. Most of these models require a large-scale labeled dataset and numerous iterations to train their parameter sets. The experimental results show that our innovative method can achieve satisfied classification results with fewer training samples. This means that in the application of RSISC, the increased workload of data annotation can be reduced manually by skilled professionals. In order to improve classification accuracy, the sum of the CNNs layer has expanded from few layers to hundred layers. Usually, the vast majority of models have huge parameters; moreover, the operation of the CNN models requires an enormity of labeled datasets for model training and powerful equipment with high-performance GPUs for notable performance improvements in operation, which severely limits the development of RSISC methods. However, in comparison with huge CNNs, the proposed method is a compact, lightweight, and efficient RSISC model with fewer parameters. On the other side, the proposed method is extremely expensive and time-consuming, due to the limitations of cross-platform libraries for the differentiable programming of quantum computers. In the future, the method should be improved in quantum circuit simulators with support for automatic differentiation, just-in-time compiling, hardware acceleration, and vectorized parallelism. Especially when the quantum circuit size or the batch dimension is large, the new platform can enable acceleration in quantum circuit simulation.

We should note, however, that our proposed method should be viewed as a new research idea for RSISC, and not be viewed as a replacement for classical pre-trained CNN models. More importantly, a hybrid classical–quantum transferring CNN provides invaluable intuition for constructing a quantum-physics-based DL method. Finally, in terms of future work, we will study process suitability and efficiency assessment in cross-domain RSISC.

## Figures and Tables

**Table 1:** List of acronyms.

RSI	Remote Sensing Image
CNN	Convolutional neural network
RSISC	Remote sensing image scene classification
RS	Remote sensing
DL	Deep learning
NN	Neural network
HHL	Harrow Hassidim Lloyd
VQE	Variational quantum eigensolver
QAOA	Quantum approximate optimization algorithm
PQC	Parameterized quantum circuit
NISQ	Noisy intermediate-scale quantum
VQC	Variational quantum circuit
QNN	Quantum neural network
MBS	Many-body system
CQ	Classical–quantum
QC	Quantum–classical
QQ	Quantum–quantum
CC	Classical–classical
QE	Quantum encoding
IQP	Instantaneous quantum polynomial
ILSRC	ImageNet Large Scale Visual Recognition Challenge
AID	Aerial Image dataset
OA	Overall accuracy
BoVW	Bag-of-Visual-Words
SIFT	Scale-invariant feature transform
SVM	Support vector machine

**Table 2:** Overall accuracy (%) of different training:test ratios on the EuroSAT dataset classification (training ratio = 10% and 20%).

Method (%)	10/90	20/80	Number of Parameters
BoVW (SVM, SIFT, k = 10)	54.54	56.13	_
BoVW (SVM, SIFT, k = 100)	63.07	64.80	_
BoVW (SVM, SIFT, k = 500)	65.62	67.26	_
CNN (two layers)	75.88	79.84	50.5 K
ResNet-50	75.06	88.53	25.6 M
GoogleNet	77.37	90.97	6.8 M
QCNN	93.65	94.72	18 M
CNN-QNN	94.23	95.07	17 M
**Ours**	**95.81**	**96.62**	**21.28 M**

## Data Availability

Not applicable.
